# Supplementary material for: Translational Studies Reveal the Divergent Effects of Simtuzumab Targeting LOXL2 in Idiopathic Pulmonary Fibrosis
Source: Fibrosis (Hong Kong). Author manuscript; Available in PMC 2024 Jun 13. (PMC11175361; doi:10.35534/fibrosis.2023.10007)
Supplement: Supplementary Information [file NIHMS1991821-supplement-Supplementary_Information.pdf]

Translational Studies Reveal the Divergent Effects of Simtuzumab Targeting LOXL2 in Idiopathic Pulmonary Fibrosis

Supplementary Information

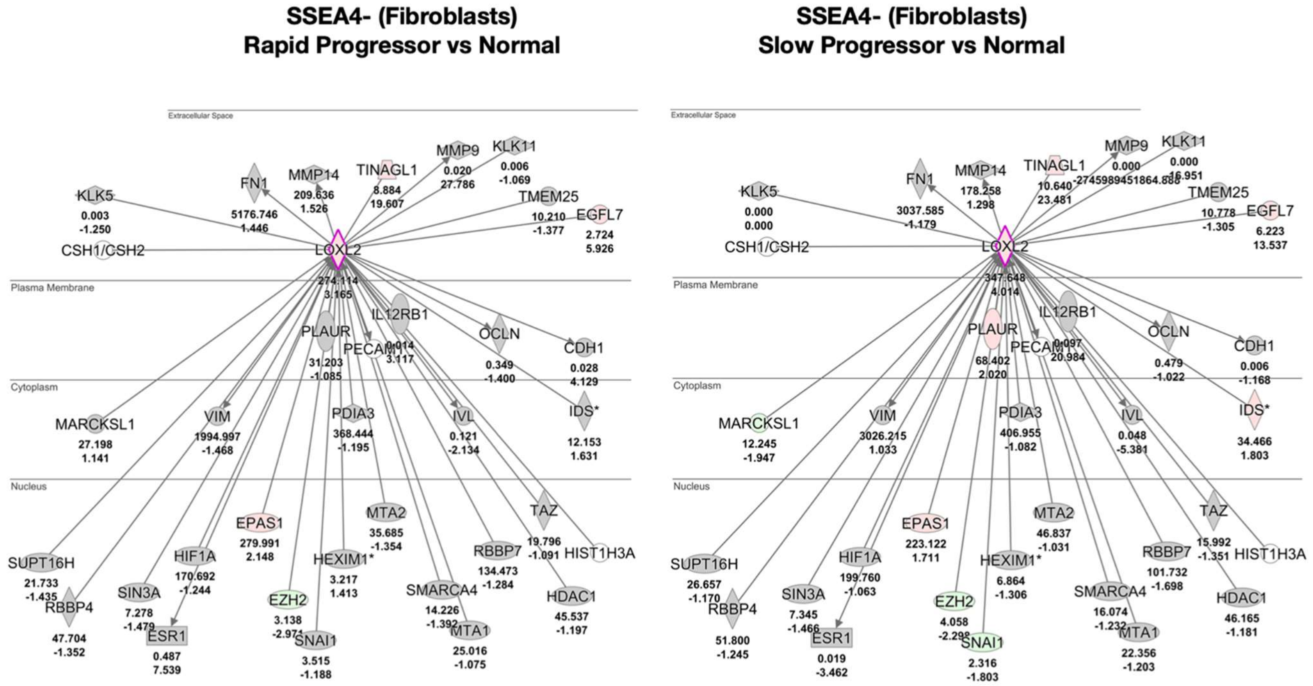

Figure S1. LOXL2 interaction network on sorted SSEA4- fibroblasts in Slow and Rapid-IPF progressors versus Normal cells. The mean FPKM counts and fold-change for interaction molecules from slow-IPF and rapid-IPF SSEA4- fibroblasts vs Normal is shown below each gene.

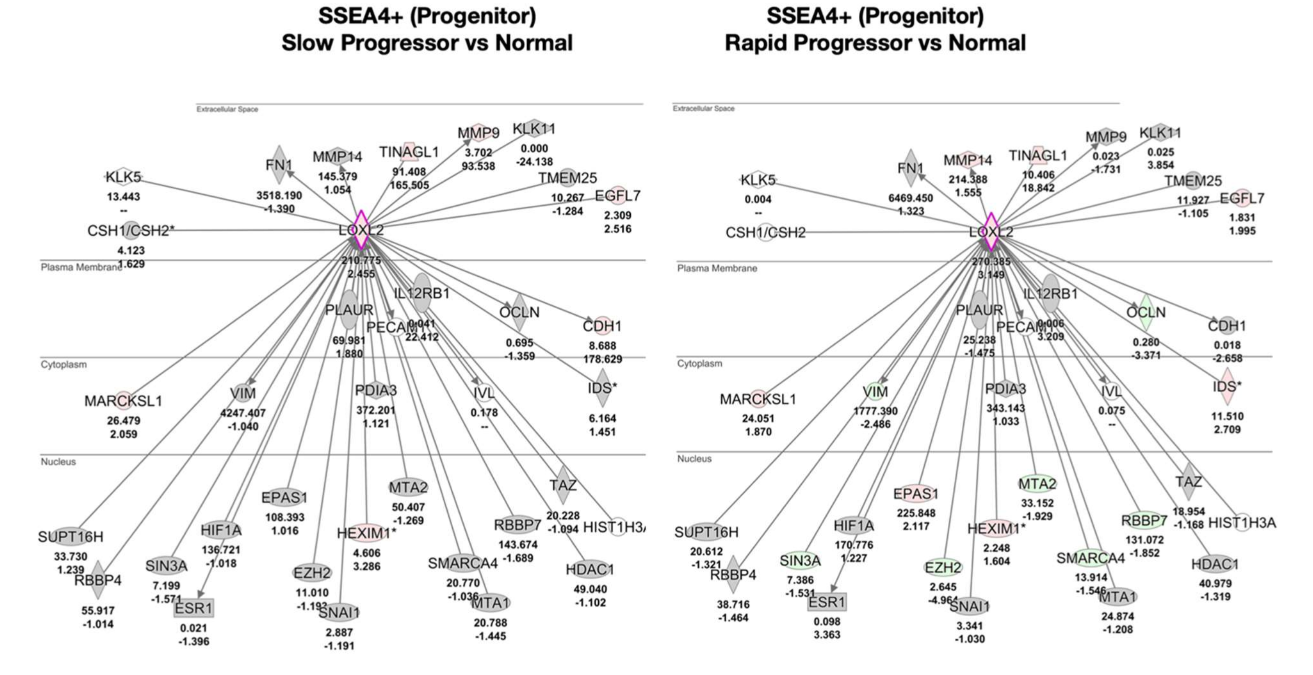

Figure S2. LOXL2 interaction network on SSEA4+ progenitor cells in Slow and Rapid-IPF progressors versus Normal cells. The mean FPKM counts and fold-change for interaction molecules from slow-IPF and rapid-IPF SSEA4+ progenitors vs Normal is shown below each gene.

Table S1. Mean FPKM counts and fold-increase expression of LOXL2 in normal vs slow-IPF and normal vs rapid-IPF SSEA4+ progenitors (a) and SSEA4− fibroblasts from previously analyzed publicly available dataset GSE103488.

**Cell type**	**LOXL2 expression**	**FPKM (Mean Counts)**	**Fold-increase (vs Normal)**
SSEA4- **Fibroblasts**	Slow Progressor vs Normal	347.64 vs 86.60	4.014
SSEA4- **Fibroblasts**	Rapid Progressor vs Normal	274.11 vs 86.60	3.165
SSEA4+ **Progenitors**	Slow Progressor vs Normal	251.94 vs 85.84	2.45
SSEA4+ **Progenitors**	Rapid Progressor vs Normal	270.38 vs 85.84	3.14

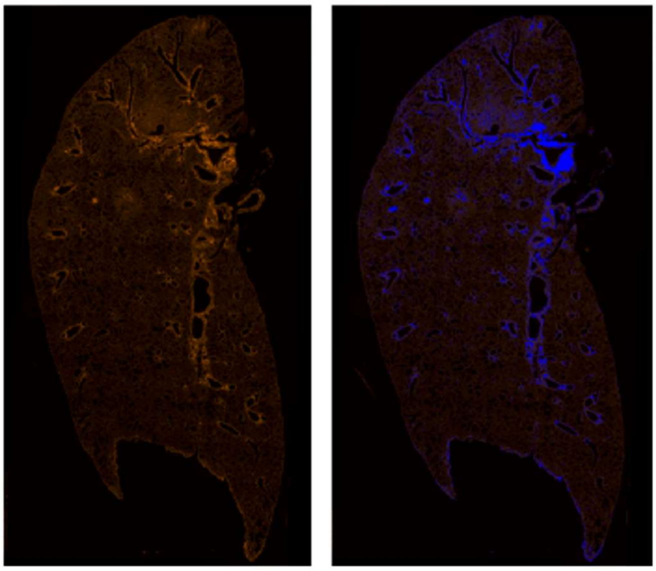

Figure S3. Mask for Picrosirius quantification.
